# Passion Fruit Seeds as a Functional Ingredient in Snack Bars: A Nutritional and Sustainable Approach

**DOI:** 10.3390/foods14111857

**Published:** 2025-05-23

**Authors:** Kezban Esen Karaca Çelik, Reyhan Irkin, Sema Çarıkçı, Simge Sipahi, Selinay Yakar, Cem Yaman, Ece Öneş

**Affiliations:** 1 Department of Nutrition and Dietetics, Faculty of Health Sciences, Izmir Demokrasi University, Izmir 35140, Türkiye; esen.karaca@idu.edu.tr (K.E.K.Ç.); reyhan.irkin@idu.edu.tr (R.I.); selinay.yakar19@gmail.com (S.Y.); cemoyamano1@gmail.com (C.Y.); 2 Department of Environmental Protection Technologies, Vocational School, Izmir Demokrasi University, Izmir 35140, Turkey; sema.carikci@idu.edu.tr; 3 Department of Nutrition and Dietetics, Faculty of Health Sciences, Acibadem Mehmet Ali Aydinlar University, Istanbul 34752, Türkiye; ece.ones@acibadem.edu.tr

**Keywords:** byproduct valorization, *Passiflora edulis*, snack bar, antioxidants, food waste, piceatannol, phenolic compounds

## Abstract

Passion fruit (*Passiflora edulis*) is consumed worldwide, and its processing generates a substantial amount of waste, particularly from the seeds and peels. This study investigated the potential of valorizing passion fruit seeds by adding them to high-fiber snack bars. Seed-enriched snack bars were evaluated for their sensory qualities, antioxidant activities, and nutritional compositions. Seed addition markedly increased the dietary fiber content (from 4.17% to 5.66%), fat content (from 15.02% to 19.63%), and antioxidant activity (e.g., 83.38% vs. 82.47% DPPH inhibition at 50 ppm) compared to the control. This was mainly due to the presence of piceatannol, a potent bioactive molecule. The overall phenolic content decreased from 90.11 to 65.37 mg GAE/100 g, suggesting intricate matrix interactions. The control bars exhibited a higher overall acceptability score, whereas the seed bars remained within the acceptable sensory range and required only minor texture adjustments. Microbiological analyses confirmed that both formulations retained their safety for 7 days at +4 °C, with appropriate levels of yeast and total viable count and no mold growth. These results suggest that passion fruit seeds have the potential to be used as a sustainable functional food ingredient. Further research is warranted to improve the sensory qualities and shelf-life stability.

## 1. Introduction

Since ancient times, people have used plants as a source of medicinal remedies. Tropical fruits are among the most famous types of fruits owing to their antioxidant and anti-inflammatory activities [1]. Compared with other tropical fruits, such as mangoes and guavas, passion fruit (*Passiflora edulis Sims*) has gained less attention despite being consumed worldwide [2,3]. It is the third most important tropical fruit after pineapple and mango [4]. It is cultivated in the Neotropical region, with Brazil being the largest producer and consumer in the world [5]. The purple species *P. edulis f. edulis* and the yellow species *P. edulis f. flavicarpa* Degener are the foundations of commercial passion fruit production. Purple passion fruit has higher levels of calcium, fiber, vitamin C, and vitamin A, whereas yellow passion fruit has higher levels of water and comparatively less nutritious components [6]. The components and potential health benefits of each part of the *Passiflora* plant—pulp, peel, seeds, and bark—have been investigated [7]. The edible portion of this fruit has been found to be effective in treating alcoholic liver disease [8]. Furthermore, it has been shown that *P. edulis* peel extract, with its dietary fiber- and functional component-rich structure, exerts anti-hypotensive effects [7] and hypoglycemic effects [9] as well as promotes metabolic improvement [10]. Meanwhile, *P. edulis* bark possesses anti-obesity properties [11].

Despite the global popularity of passion fruit, the main driving factor for its cultivation is juice production [12]. It has a juicy, soft-to-firm interior that is filled with seeds [13]. In juice-making, only the pulp of the passion fruit is utilized; the discarded parts, such as leaves, peels, bagasse, and seeds, represent 70% of the byproducts and eventually produce thousands of tons of waste annually [14]. This waste is rich in bioactive substances, such as flavonoids (e.g., vitexin), phenolic compounds (e.g., gallic acid, piceatannol, and neochlorogenic acid), and carotenoids (e.g., β-carotene and lutein). The recovery of these substances can improve diet quality and address waste disposal issues, thereby providing a nutritious and sustainable solution [2].

Among the byproducts, seeds come second after peels, both of which have important nutritional value. Peels, which are a major waste contributor, have high levels of pectin, potassium, and calcium, making them an excellent source of fiber [12]. The second byproduct, tiny, oval, black, flattened seeds, provides nutrients essential for embryonic development and is rich in lipids (especially unsaturated fatty acids, such as oleic and linoleic acids), starches, proteins, and minerals. Thus, they are attracting attention as valuable nutritional and functional materials [7,12,15]. Along with the various recipes that can be used to make salads, sauces, or as a topping, other potential routes of utilization can still be studied [13].

Snack bar consumption is an increasing global trend, which is largely influenced by lifestyle changes among consumers and their health awareness [16]. Snack bars are commonly consumed by customers who require a rapid source of energy, owing to the lack of time to eat enough [17]. They serve various purposes, such as increasing the intake of energy, protein, carbohydrates, lipids, vitamins, and minerals. Nutrition bars, which include numerous ingredients to appeal to a broad spectrum of consumer groups, are popular healthy snacks among young people, athletes, vegans, dieters, and others. Cereals, nuts, nut pastes, seeds, and dried fruits can be consumed in place of junk food in these nutritious snacks [18]. Peels, seeds, and pomace, which are byproducts of fruit and vegetable processing, were found to have a high content of antioxidant dietary fiber and to possess nutritional and physicochemical qualities that are valuable in the food industry. These fibers are frequently linked to bound phenolic chemicals, which preserve the fiber’s structural and functional qualities while promoting antioxidant activity [19]. As previously mentioned, snack bars enriched with *P. edulis* seeds could be a great option for improving the quality of the diet.

This study aimed to assess the nutritional and functional qualities of *P. edulis* seeds and explore their potential for sustainable food production and waste valorization. The hypothesis of this study is that the incorporation of passion fruit (*P. edulis*) seed into snack bar formulations would substantially alter their nutritional composition, antioxidant capacity, and total phenolic content compared with control formulations. Furthermore, this study aimed to develop a seed-enriched snack bar and evaluate the impact of seed addition on its functional properties and sensory characteristics.

## 2. Materials and Methods

### 2.1. Materials

For the snack bar production, the ingredients were carefully selected, taking into account their compatibility with each other in terms of color, smell, texture, and taste. Purple passion fruit (*Passiflora edulis f. edulis*), dates, walnuts, peanut butter, oats, and carob extract were obtained from local markets in İzmir, Türkiye, and cinnamon and coconut oil were obtained from a herbalist’s shop. All analytical-grade chemicals and reagents were purchased from Sigma-Aldrich (Steinhein, Germany). All raw materials were stored at 4 °C until further use.

### 2.2. Preparation of P. edulis Seeds

**Figure 1:**
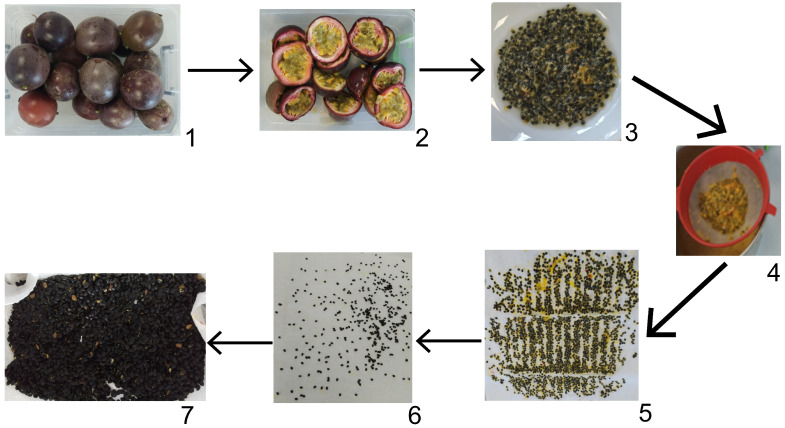
Preparation process of *P. edulis*. (1) Selection of fruits. (2) Cutting of fruits. (3) Separation of seeds. (4) Rinsing of seeds. (5) Drying preparation. (6) Drying of seeds. (7) Grinding of seeds.

Purple passion fruits were sorted, thoroughly washed, and cut in half. The pulp was removed using a spoon, and then the seeds were separated using a strainer. The seeds were submerged in running water, and excess water was removed using blotting paper. The outermost mucilage layer of the seeds was removed during the washing process. Subsequently, the seeds were laid out on blotting paper and dried in a laboratory drying oven at 60 °C for 24 h to ensure standard sample preparation [20]. After drying, the seeds were powdered using a high-speed shredder [13]. Seed preparation is illustrated in Figure 1. 

### 2.3. Preparation of Snack Bars

**Figure 2:**
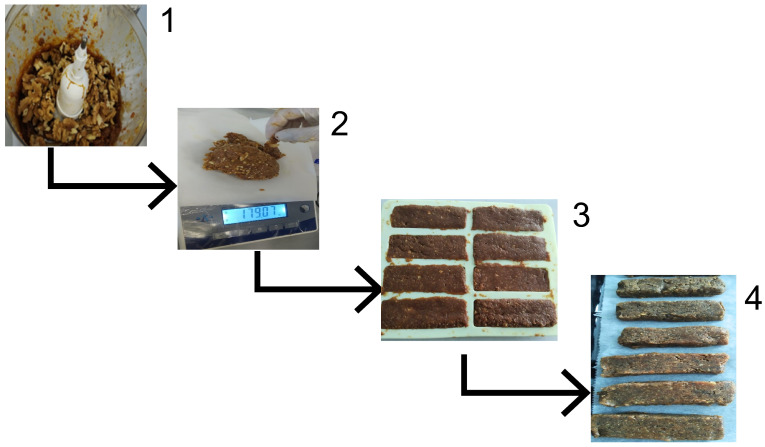
Preparation steps for the snack bars. (1) Blending of dates until a paste-like consistency was obtained. (2) Weighing of the bar dough. (3) Placement of the dough in molds. (4) Baking and cooling of the bars.

Snack bars were prepared using the method described by Tokpunar et al., with slight modifications [17]. After sorting, washing, and removing the seeds, the bars were immediately formulated. Microbiological safety was ensured by immediately incorporating the seeds into the bar mixture. The dates and hot water, sufficient to cover the dates, were mixed in a food processor until a past-like consistency was achieved. All the remaining ingredients were then added to achieve a homogeneous consistency. The prepared bar dough was weighed and divided into control bars (CBs) and seed bars (SBs). To standardize the weight of the snack bars, packaged snack bars were used as weight samples. The bar dough was placed in molds at a standardized weight of approximately 30 g per bar. SBs were added to finely ground passion fruit seeds, whereas CBs were kept as they were. The amount of passion fruit seed powder incorporated into the bar formulations was determined based on the results reported by Sano et al. (2011) and Uchida-Maruki et al. (2015), aiming to achieve a functionally relevant piceatannol intake [21,22]. Accordingly, 7.5 g of passion fruit seed powder was added to each 30 g bar, corresponding to 25% (*w*/*w*) of the total formulation. Subsequently, the bars were baked for 15 min in an oven at 150 °C. Microbial reduction in the seeds was achieved during subsequent baking. The baked bars were removed from the molds after they reached room temperature. All bars were prepared in a single production session under identical conditions. Analytical measurements for each group were performed in triplicate, and the results were expressed as mean ± standard deviation (SD) to reflect the variability. To maintain microbiological stability during the analytical step, the bars were briefly stored at +4 °C after being tightly wrapped in aluminum foil. In the first assessment, product safety was guaranteed even if the storage conditions did not represent normal market circumstances. The preparation of the snack bars is shown in Figure 2.

### 2.4. Preparation of the Extracts

Samples of snack bars (200 mg) were extracted using 3 × 10 mL of ethanol. The obtained extracts were then combined and purified via filtration through membrane filters with a pore size of 0.2 mm. Ethanol was removed using a rotary evaporator (BUCHI R100 Rotary Evaporator, Flawil, Switzerland), and then the remaining extract was dissolved in 2 mL of ethanol (96%). To determine the DPPH antioxidant scavenging activity and total phenolic content, extracts were prepared using 50% (*w*/*v*) ethanol. Next, the prepared extracts were stored at +4 °C until use for the determination of the total phenolic content and for DPPH assays [23].

### 2.5. Chemical Analysis

The chemical compositions of the samples were analyzed using standardized methods, including Turkish standards (TS) and international methods. The carbohydrate and energy contents (kcal/100 g) were determined using the TS 11729 method; fat content, the TS 2664 method; protein content (N × 6.25), the TS 1620 method; ash content, the TS 2131 ISO 928 method; cellulose content, the TS 6932 method; moisture content, the TS 541 method; and dietary fiber content, the AOAC 985.29 method.

To examine the mineral content, calcium, magnesium, zinc, sodium, potassium, and iron were selected and quantitatively analyzed using ICP-MS. Calcium, magnesium, zinc, sodium, and potassium were determined using the TS 3606 method, and iron was determined using the NMKL 186 international method. Each analysis was performed in triplicate.

### 2.6. Determination of Dry Matter

Dry matter was determined using the Association of Official Analytical Chemists (AOAC) 934.06 method [24]. The dry matter of all the bar samples in the drying containers with added sea sand was kept in an oven at 105 °C for a certain number of hours, cooled in a desiccator, and weighed on a precision balance was separately weighed. According to the difference in precision weighing, the dry matter (%) was determined using the gravimetric method.

### 2.7. Determination of the Total Phenolic Content

The total phenolic content of the snack bars was determined to demonstrate the amount of phenolic content in the snack bars and seeds. The total phenolic content of the snack bars, which was extracted using 96% ethanol, was quantified via the Folin–Ciocalteu assay, as described by Singleton and Rossi (1965) [25], following the modifications made by Gündüz et al. [23]. First, 0.5 mL of the sample extract was placed into a test tube, and 2.5 mL of the Folin–Ciocalteu reagent (0.2 N) was added. Instead of the sample, 0.5 mL of ethanol was used as the blind solution. Next, the mixtures were vortexed for 15 s, and 2 mL of 7.5% (*w*/*v*) sodium carbonate was added. The mixture was then kept in the dark for 90 min. Finally, the absorbance values of the solutions were read at 720-nm wavelength (Shimadzu UV-1280, Kyoto, Japan) against a blank. A standard calibration curve was prepared using gallic acid at concentrations of 0, 50, 100, 125, 150, and 200 mg/mL. Absorbance was measured at 720 nm. The standard curve exhibited linearity with the regression equation y = 0.0114x − 0.0192 and a correlation coefficient of R^2^ = 0.986. The total phenolic content of the samples was calculated using the equation: The total phenolic content of the samples was determined by calculating the mean absorbance values in triplicate. The results are expressed as mg gallic acid equivalent (mg GAE) per 100 g of sample.

### 2.8. Determination of the Antioxidant Activity

Antioxidant activity was assessed using a commonly used method based on the inhibition of 2,2-diphenyl-1-picrylhydrazyl (DPPH) radicals, modifying the method outlined by Özer et al. [26]. Among the available methods, the DPPH assay was selected due to its simplicity, sensitivity, and widespread use for measuring radical scavenging activity in food matrices. For this purpose, 4 mg of DPPH radical was weighed and dissolved in 100 mL of 98% ethyl alcohol. The mixture was stirred in the dark for 30 min using a magnetic bar. Then, 40, 100, 200, and 400 μL of the samples were extracted, and a total volume of 800 μL was achieved by adding ethyl alcohol. The total volume was adjusted to 4000 μL by adding 3200 μL of the prepared DPPH radical solution. Ethyl alcohol was used as a blind solvent. The mixtures were stirred using a vortex device for 15 s and then stored at room temperature in the dark for 30 min. The absorbance of the samples was measured in triplicate at 517 nm using a UV spectrophotometer (Shimadzu UV-1280, Kyoto, Japan). Subsequently, the mean absorbance values were calculated, and the DPPH radical scavenging activity of the samples was calculated as a percentage inhibition value using the equation DPPH radical scavenging activity (%) = [(A_0_ − A_1_)/A_0_] × 100, where A_0_ denotes the absorbance of the control, and A_1_ represents the absorbance of the sample extract.

### 2.9. Microbiological Analysis

For microbiological analysis, a 10-g bar sample was mixed with 90 mL of sterile dilution liquid (8.5 g NaCl/l [*w*/*v*]) and homogenized using a stomacher (Stomacher 80, Seward Medical, London, U.K.) at low speed for 2 min at room temperature. Subsequently, serial dilutions were prepared from the 1/10 dilution obtained [27]. Microbiological analysis was conducted on the bars, which included the determination of the total viable count (TVC) and the total yeast and mold count. TVCs were determined using plate count agar after incubation for 48 h at 35 °C. The results were expressed as log CFU/g [28]. For the total yeast and mold count, potato dextrose agar (PDA) was used as the medium. Under aseptic conditions, PDA was poured into Petri dishes, and the prepared dilutions were inoculated using the spread-plate method. The Petri dishes were incubated at 25 °C for 7 days. Yeast colonies were counted after 3 days, whereas mold colonies were counted after 7 days, and the results were expressed as CFU/g [23].

### 2.10. Sensory Evaluation

Sensory evaluation of the snack bars was conducted using a “5-point hedonic scale”. A total of 12 individuals were included in the evaluation. A scale of “1: I did not like it at all, 2: I liked it less, 3: neither like nor dislike, 4: I like it a little bit, 5: I liked it very much” was used to score the color, flavor, taste, appearance, texture, and overall acceptability criteria. The participants were notified of the form beforehand. The inclusion criteria were as follows: participants who were willing to participate in the study, aged between 18 and 45 years, free of allergies or intolerances, not taking regular medications, not eating a regular diet, not smoking, not hungry or extremely full at the time of tasting, able to spare time for the evaluation, and interested in the subject [23].

### 2.11. Statistical Analysis

The acquired results are expressed in tables as mean ± SD. The data from the sensory evaluations are also shown graphically. Statistical analyses were performed using IBM SPSS Statistics 25.0 software. A paired *t*-test was employed to compare the results of the CBs and SBs, with *p* = 0.05 indicating statistical significance. All analyses were performed in triplicate.

## 3. Results

### 3.1. Chemical Composition

To better understand the differences in the nutritional profiles of the snack bars, which reflect the quality and proportion of the components used, the relationship between their nutritional compositions was evaluated. The chemical compositions of the SBs and CBs are listed in Table 1.

*Passiflora* seeds independently showed significantly high contents of fat (19.63 ± 1.39 g/100 g), cellulose (7.84 ± 0.35 g/100 g), and dietary fiber (5.66 ± 0.68 g/100 g), whereas the CBs showed a statistically high content of carbohydrate (49.92 ± 2.51 g/100 g) with low energy level (379.8 ± 2.20 kcal/100 g). No significant differences were observed between the snack bars in terms of protein, ash, and moisture content. The energy content of the CBs was within the acceptable range of 300–450 kcal/100 g provided by the date bars [29]. The energy content of the CBs in the present study was higher than that in previous studies [30,31], but it was very similar to that observed by İbrahim et al. [32]. The energy content of the SBs in the present study was higher than that of the CBs (*p* < 0.05). The increase observed with the addition of *P. edulis* seeds was also comparable to the results of the study by İbrahim et al., who reported that the addition of walnuts increased the energy content to approximately 707–413 kcal/100 g [32]. The increased energy content in the SBs can be attributed to the change in the food matrix caused by the addition of *P. edulis* seeds. For a snack bar to be used as a meal replacement, it should provide 300 kcal per serving, which was achieved by our SBs and CBs [30].

The carbohydrate content of the CBs was 49.92 ± 2.51 g/100 g, which is below the general range observed in the literature [31,32,33,34]. When *P. edulis* seeds were added, the carbohydrate content of the snack bar decreased to 49.92 ± 2.51 g/100 g (*p* < 0.05). This difference could be attributed to the method employed in the preparation of the bars (providing lower carbohydrate content but higher fat content to the ingredients), according to the different date varieties, ripening stage, or geographical conditions under which the dates were harvested [29,35].

The base ingredient of our snack bars, which are dates, typically contains low levels of fat, with varying quantities in different cultivars [29]. The CBs were found to have fat content of 15.02 ± 1.06 g/100 g, which is similar to the findings of Aljaloudi et al. [30]. However, in line with the method followed in snack bar production, with the addition of walnuts, peanut butter, and coconut oil, the fat content of the CBs was also higher than that reported in some previous studies [31,32]. In comparison, the fat content of the SBs was higher (19.63 ± 1.39 g/100 g, *p* < 0.05), which can be attributed to the fat content of the seeds. *P. edulis* seeds provide approximately 12–33 g of fat per 100 g dry weight [7]. The key component of *P. edulis* seeds is considered to be lipidic composition, as linoleic, linolenic, oleic, palmitic, and stearic acids [15]. Purple passion fruit seed oil was found to mainly consist of unsaturated fatty acids (85% and 97%), with linoleic acid demonstrating the highest proportion (70% and 36%), and having an unsaturated/saturated fatty acid ratio of 8.12 [15,36]. Furthermore, the high polyunsaturated fatty acid content of the seed-enriched bars may increase the risk of lipid oxidation during prolonged storage, which may compromise their flavor, texture, and nutritional value. To reduce oxidative deterioration, future formulations could benefit from the use of natural antioxidants and protective packaging techniques [37,38]. In addition to fatty acids, other minor components, such as sterols and tocopherols, have been identified in purple passion fruit seed oil. A total sterol content of 2.09–3.33 mg/g in seed oil has been reported, mainly containing β-sitosterol (41.5–42.5% of the total phytosterol content), stigmasterol (30.9–41.7% of the total phytosterol content), and campesterol (11.1–13.5% of the total phytosterol content) [15]. A similar increase in fat levels was observed when nuts and seeds were added to the bars [29].

In snack bars, the protein content typically ranges from 5% to 25% [29,30,32,39,40]. In our study, the protein content of both bars was within the range. Although purple passion fruits have low protein content, their seeds are the richest part in terms of protein content (13.2% of dry weight) [15]. No significant difference was observed in protein content with the addition of *P. edulis* seeds, which could be related to the incorporation ratio of a single seed.

The amount of ash in a dietary source is generally used as an indicator of its mineral content [31]. As regards the ash content, no significant differences were observed among the bars in this study (CBs: 1.82 ± 0.15 g/100 g, SBs: 11.80 ± 0.15 g/100 g). These values are lower than those in previous studies [30,31,34] but higher than those observed by Munir et al. and Nadeem et al. [33,41]. A similar lack of significance was observed by Eid et al. after the fortification of date bars with different ratios of *Moringa oleifera* [31].

The moisture values (CB: 16.87 ± 1.49%, SB: 16.56 ± 1.46%) obtained in this study were higher than those reported by [42] but lower than those reported by [30,31,33,34,41]. Moisture content is a critical parameter for shelf-life and texture; therefore, low moisture levels, consistent with our results, can markedly improve shelf-life stability by limiting microbial development [29]. Although the detected moisture content of the bars was within an acceptable range, it may have contributed to elevated water activity levels, which could have an impact on microbiological stability and shelf-life over time. Water activity measurements and the formulation or packaging of solutions to reduce this risk should be investigated in future research.

Seeds are good sources of dietary fiber [43]. The bar sample enriched with *P. edulis* seeds (5.66 ± 0.68) had a higher dietary fiber content than the CBs (4.17 ± 0.50) (*p* < 0.05). This discrepancy is attributed to the high fiber content of the seeds. The dietary levels of the SBs and CBs were within the range of 2–10%, as reported in previous studies [17,29,33,34]. As products with a fiber content of 3 g/100 g are considered to be “fiber source,” our CBs and SBs can qualify as fiber sources [32]. Dietary fiber includes both insoluble and soluble fractions. Cellulose was presented separately to demonstrate the specific contribution of insoluble fiber associated with seed incorporation in the diet. The majority of the dietary fibers observed in seeds are insoluble. Delvar et al. detected 521 mg/g of dry-weight insoluble fiber in *P. edulis* seeds [44]. The increase in dietary fiber content in our study may be related to the cellulose content of the seeds. When the cellulose levels of the bars were examined, a significant increase was observed with the addition of *P. edulis* seeds (*p* < 0.05). Passion fruit seeds are a good fiber source and a useful low-calorie bulk ingredient in food applications. With their fiber- and cellulose-rich compositions, the seeds may treat intestinal peristalsis by increasing fecal size and reducing transit time. In addition, researchers have found that this rich structure may help regulate postprandial serum glucose levels and be prospectively used as low-calorie bulk components for dietetic snacks and fiber enrichment [3]. The benefits of fiber for digestive health, energy balance, cancer, heart disease, and diabetes highlight the need for everyday diets to contain more dietary fiber [45]. The incorporation of *P. edulis* seeds into date bars led to a substantial increase in pH (*p* < 0.05). Silva et al. reported that the pH value of *P. edulis* seeds was 6.36 ± 0.08, which could be related to the slight increase in the pH value [43]. The values we obtained were lower than those reported by Eid et al. and Silva et al. but higher than those reported by Tokpunar et al. [16,17,31].

The mineral content of the snack bars is presented in Table 2. No statistical difference was observed between the SBs and CBs in terms of the mineral content. This suggests that the mineral profile of the bars was not markedly affected by the addition of *P. edulis* seeds to their composition. This result could be attributed to the use of only one ratio of fortification when obtaining the SBs. The examination of the mineral content of SBs prepared using different ratios can help achieve a comprehensive evaluation.

### 3.2. Functional Properties

The phytochemical composition of *P. edulis* is significantly affected by various factors, including cultivar, climate, agricultural management practices, and the lack of standardization of the analytical methods employed [15,34]. As antioxidants, phenolic compounds can substantially contribute to overall antioxidant activity. The available concentration of a single antioxidant component or the possible synergistic interaction between different plant ingredients can determine the activity [34]. The effect of the addition of *P. edulis* seeds on the total phenolic content of the bar samples is illustrated in Table 3.

The total phenolic content of *P. edulis* seeds in this study was higher than that reported previously [3,46,47]. Phenolic acids, including ferulic, gallic, chlorogenic, and caffeic acids, account for a considerable portion of the phenolic chemicals found in seeds. Although these seeds contain phenolic compounds, they are not the richest components [15]. Accordingly, SBs exhibited lower total phenolic content than CBs. This could be related to the ratio of fortification used or the method employed to assess the total phenolic content. In a study conducted by dos Reis et al., the total phenolic content of purple passion fruit seeds (3.26 mg GAE/g) was lower than that of yellow and orange passion fruit seeds (3.46 and 4.29 mg GAE/g, respectively), and the researchers concluded that the method employed was not specific for the remaining phenolic compounds found in the plant [48]. In our study, a similar situation was observed; therefore, different approaches to quantify the total phenolic content would be valuable for understanding the phenolic profile of the analyzed samples.

**Figure 3:**
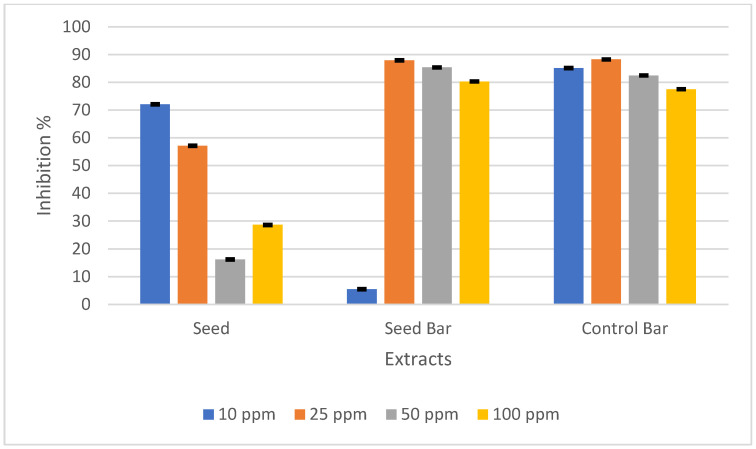
DPPH radical scavenging activity (%) of *Passiflora edulis* seeds, seed-enriched snack bars, and control bars at different concentrations (10, 25, 50, and 100 ppm). The results are expressed as the mean ± standard deviation (*n* = 3).

Santana et al. subjected *P. edulis* seeds to various extraction conditions and investigated the association between the component concentration and antioxidant activity in each scenario. The findings indicated a positive relationship between the phenolic compound concentration and antioxidant activity of the extracts, suggesting that phenolic compounds play a pivotal role in antioxidant activity [49]. To measure the antioxidant activity, the DPPH radical scavenging activity was used. The antioxidant activities of *P. edulis* seeds, SBs, and CBs prepared at concentrations of 10, 25, 50, and 100 ppm are presented in Figure 3.

The antioxidant activity of the *P. edulis* seed in this study is consistent with that in previous studies [7]. Although the total phenolic content was lower, the antioxidant activity was higher in SBs than in CBs. This contrast may have stemmed from the largest contribution of phenolics, piceatannol, which exhibits rich antioxidant activity, even in small amounts [7,50]. Piceatannol, a polyphenolic stilbene phytochemical, is recognized as a hydroxylated analog of resveratrol that demonstrates resveratrol-like activity [51]. First reported by Matsui et al., *P. edulis* seeds have a very high level of piceatannol, which is over 50 times higher than that of another rich source, such as grapes [50]. This substance possesses anti-oxidative, anti-inflammatory, anti-cancer, anti-atherogenic, estrogenic, and antibacterial properties. This compound can also enhance the physicochemical properties and functionality of the product [2].

Morais et al. reported that passion fruit seeds have a high total phenolic content and the highest antioxidant capacities in the FRAP assay compared to pulp, as well as raw, oven-dried, and lyophilized peels. Nevertheless, they demonstrated the lowest IC_50_ value (DPPH) of 49.71 mmol, which is one level lower than that of pulp and peels [52]. The low antioxidant activity observed could also be attributed to the analysis method employed. For a better understanding, further analysis of the antioxidant activity of both seeds and SBs should be conducted.

### 3.3. Microbiological Analysis

Appropriate culture media were prepared to determine the total yeast and mold count and TVC in the snack bar samples. Observations were performed on days 1, 3, and 7. Microbiological activity is a key component of food product quality and shelf-life stability. In addition to the nutritional content, texture, and sensory appeal of the bars, it is essential for the health and safety of consumers [31]. Table 4 presents the microbial counts of CBs and SBs.

All samples exhibited an increase in TVC and yeast counts over the storage period, with fluctuations. The increase in yeast counts over time in both bars was part of the natural process. Yeast proliferation was expected during production and storage. The increase in TVC can be associated with an increase in yeast count. While not applied in the current study, modified atmospheric packaging could be recommended to limit yeast growth in future formulations [53]. The slightly elevated yeast levels in the SBs compared to the CBs could be attributed to the natural microbial load associated with fruit seeds. Moreover, the fermentable carbohydrates in the bar matrix may have provided favorable conditions for yeast. However, the absence of mold growth during refrigeration suggests that microbial spoilage was effectively prevented under the applied conditions [54]. An increase in the TVC as well as the yeast and mold count was observed in a similar trend in previous studies, in different storage times ranging from 70 to 90 days; such an increase was associated with the change in water activity during storage [53,55,56]. Water activity is a crucial indicator of microbiological stability; therefore, it should be monitored throughout the storage period at different time points, aligned with microbiological analyses. Although the results obtained from the CBs and SBs remained stable and safe for consumption until the conclusion of the 7-day period, as the total bacterial count was within the statutory permissible range (6 log CFU), the fluctuations in the microbial count may have been caused by variations in water activity during storage [18]. The SBs were found to be rich in cellulose, which Chau et al. reported as a component with a high water-holding capacity [3]. This capacity of cellulose may be associated with high water activity, resulting in increased microbiological activity. Longer storage durations would provide a more comprehensive understanding of microbiological development in the bars.

### 3.4. Sensory Evaluation

Consumer acceptance and satisfaction with nutrition bars are greatly influenced by the bars’ flavor and texture [57]. In addition to their beneficial impacts on nutritional content, adding taste-intense components such as nuts or dried fruits can enhance the acceptance of date bars in terms of taste, flavor, odor, and color [29].

**Figure 4:**
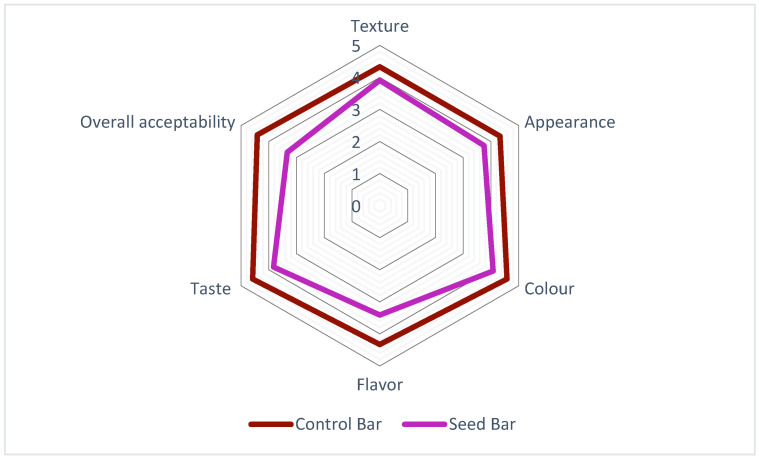
Sensory analysis of the bar formulations with *P. edulis* seeds.

Both CBs and SBs were subjected to sensory evaluation immediately after the production process was completed. In most studies, the key parameters used for sensory evaluation include appearance, color, flavor, taste, texture, and overall acceptability. These parameters were also used in the present study to better understand how consumers perceive these products. The results of the sensory evaluation of the SBs and CBs are illustrated in Figure 4. The CBs (denoted by the red line) consistently achieved higher scores across all attributes than the SBs (represented by the purple line). Notably, the greatest differences were observed in texture and overall acceptability, for which CBs demonstrated superior results. The SBs exhibited scores closest to those of CB in terms of appearance and color among the analyzed factors. While the CBs maintained an overall advantage, the disparities in these specific attributes were relatively small. In addition, the SBs remained within the acceptable range, demonstrating their potential for improving specific sensory characteristics. Similar results were obtained in previous studies, suggesting that CBs usually achieve the highest scores, largely depending on their formulation [29,40]. Purple passion fruit seed oil was found to have high levels of alcohol, ester, ketone, and acid, which provide floral, fruity, creamy, and yogurt flavors [36]. This composition may be related to consumer preferences. Nevertheless, the differences observed in our study were less noticeable between CBs and SBs, suggesting similar sensory performances for the two formulations.

## 4. Conclusions

This study demonstrates that the nutritional and antioxidant profiles of SBs were enhanced by the addition of *P. edulis* seeds, showing their potential in the creation of functional snack bars, especially due to their high dietary fiber content and bioactive chemicals, largely confirming the tested hypothesis. However, limitations were observed during the sensory evaluation, as the SBs had poorer texture and general appeal than the CBs. These results indicate that further optimization is required to improve customer acceptability while preserving the functional and nutritional benefits of the products. Another related limitation of this study is the relatively small sample size of the sensory panel, which may affect the generalizability of the findings. Second, the assessment of long-term microbiological stability in this study was constrained by the short storage duration. The slightly higher water activity observed in the SBs than in the CBs poses challenges for extended shelf-life, warranting further research into advanced packaging solutions. As this study was designed as a preliminary study, investigations on extended shelf-life and sensory optimization were outside the scope of the current work due to time and resource restrictions within the framework of budget and time limitations. Furthermore, including additional antioxidant assays, such as ABTS, FRAP, and ORAC, in future studies could provide a more comprehensive evaluation of antioxidant capacity.

Future studies should investigate the effects of different amounts of seed fortification on the functional, sensory, and nutritional qualities of snack bars. This would provide a more thorough understanding of the best formulations for producing bars with great health benefits and high consumer acceptance. In addition, although the antioxidant activity and bioactive chemical concentration of *P. edulis* seeds have been demonstrated, the bioavailability of these compounds and their effects on health in vivo remain unknown. Lastly, to evaluate the variation in their nutritional and bioactive profiles, research on the use of seeds from various passion fruit cultivars and geographic locations may be useful. This would allow the development of standardized and universally applicable formulations. In conclusion, this study highlights the promising potential of *P. edulis* seeds for developing functional snack bars, offering a sustainable, fiber-rich, and antioxidant-enhanced alternative to conventional products.

## Figures and Tables

**Table 1:** Chemical composition of the snack bars.

Nutritional Composition	Control Bars Mean ± SD	Seed Bars Mean ± SD
Energy (kcal/100 g)	379.8 ± 2.20 ^a^	393.3 ± 2.13 ^b^
Carbohydrate (% *m*/*m*)	49.92 ± 2.51 ^a^	42.35 ± 2.13 ^b^
Fat (% *m*/*m*)	15.02 ± 1.06 ^a^	19.63 ± 1.39 ^b^
Protein (Nx6.25) (% *m*/*m*)	11.24 ± 0.75 ^a^	11.83 ± 0.79 ^a^
Ash (% *m*/*m*)	1.82 ± 0.15 ^a^	1.80 ± 0.15 ^a^
Cellulose (% *m*/*m*)	5.13 ± 0.23 ^a^	7.84 ± 0.35 ^b^
Moisture (% *m*/*m*)	16.87 ± 1.49 ^a^	16.56 ± 1.46 ^a^
Dietary Fiber (% *m*/*m*)	4.17 ± 0.50 ^a^	5.66 ± 0.68 ^b^
pH	5.59 ± 0.01 ^a^	5.62 ± 0.01 ^b^

Note: The different letters within the same row indicate significant differences (*n* = 3; *p* < 0.05).

**Table 2:** Mineral content of the snack bars.

	Control Bars Mean ± SD	Seed Bars Mean ± SD
Calcium (mg/kg)	948.92 ± 319.75 ^a^	962.17 ± 324.22 ^a^
Magnesium (mg/kg)	619.76 ± 194.05 ^a^	704.18 ± 220.49 ^a^
Zinc (mg/kg)	15.14 ± 4.18 ^a^	15.21 ± 4.20 ^a^
Sodium (mg/kg)	1410.78 ± 517.02 ^a^	1116.48 ± 409.16 ^a^
Potassium (mg/kg)	11,327.47 ± 3568.75 ^a^	13,016.36 ± 4100.84 ^a^
Iron (mg/kg)	21.63 ± 3.43 ^a^	17.95 ± 2.85 ^a^

Note: Different letters within the same row indicate significant differences (*n* = 3; *p* < 0.05).

**Table 3:** Total phenolic content of *Passiflora* seeds and snack bars.

	Total Phenolic Content Mean ± SD
Seed (mg GAE/100 g)	140.02 ± 0.85
Seed Bars (mg GAE/100 g)	65.37 ± 0.26
Control Bars (mg GAE/100 g)	90.11 ± 0.32

**Table 4:** Microbial counts of the control bar and seed bar for a period of 7 days.

	Control Bar			Seed Bar		
Measure	Day 1	Day 3	Day 7	Day 1	Day 3	Day 7
Yeast (log CFU/g)	2.66	3.00	4.36	3.28	4.30	4.58
Mold (log CFU/g)	-	-	-	-	-	-
Total viable count (TVC) (log CFU/g)	3.49	4.37	4.11	3.62	4.14	4.43

## Data Availability

The original contributions presented in the study are included in the article, further inquiries can be directed to the corresponding author.

## Abbreviations

The following abbreviations are used in this manuscript:

AOAC	Association of Official Analytical Chemists
CB	Control bar
CFU	Colony-forming unit
DPPH	2,2-Diphenyl-1-picrylhydrazyl
GAE	Gallic acid equivalent
PDA	Potato dextrose agar
SD	Standard deviation
TVC	Total viable count
